# Dissecting Genetic Network of Fruit Branch Traits in Upland Cotton by Association Mapping Using SSR Markers

**DOI:** 10.1371/journal.pone.0162815

**Published:** 2017-01-25

**Authors:** Yongjun Mei, Jiwen Yu, Angli Xue, Shuli Fan, Meizhen Song, Chaoyou Pang, Wenfeng Pei, Shuxun Yu, Jun Zhu

**Affiliations:** 1 College of Plant Science, Tarim University, Alar, Xinjiang, China; 2 State Key Laboratory of Cotton Biology, Key Laboratory of Cotton Genetic Improvement, Ministry of Agriculture, Institute of Cotton Research, Chinese Academy of Agricultural Sciences, Anyang, Henan, China; 3 Key Laboratory of Crop Germplasm Resource of Zhejiang Province, Zhejiang University, Hangzhou, China; New Mexico State University, UNITED STATES

## Abstract

Genetic architecture of branch traits has large influences on the morphological structure, photosynthetic capacity, planting density, and yield of Upland cotton (*Gossypium hirsutum L*.). This research aims to reveal the genetic effects of six branch traits, including bottom fruit branch node number (BFBNN), bottom fruit branch length (BFBL), middle fruit branch node number (MFBNN), middle fruit branch length (MFBL), upper fruit branch node number (UFBNN), and upper fruit branch length (UFBL). Association mapping was conducted for these traits of 39 lines and their 178 F_1_ hybrids in three environments. There were 20 highly significant Quantitative Trait SSRs (QTSs) detected by mixed linear model approach analyzing a full genetic model with genetic effects of additive, dominance, epistasis and their environment interaction. The phenotypic variation explained by genetic effects ranged from 32.64 ~ 91.61%, suggesting these branch traits largely influenced by genetic factors.

## Introduction

*Gossypium hirsutum* is one of the commercially grown species of cotton. In the past decades, thousands of analyses have been carried out to find what factors are important in cotton growth. Previous studies have revealed that branch traits were related to plant density, canopy structure, and photosynthetic capacity, thus influencing fiber quality and yield [1,2]. Association mapping aims to discover quantitative trait loci (QTLs) by evaluating the marker-trait associates, which influences the strength of linkage disequilibrium between genotypes and phenotypes across a population [3]. Since association mapping has been used in many researches related to complex disease and agronomic traits, it becomes an effective way to dissect the genetic basis of complex traits. Compared with linkage analysis, association mapping has three advantages: no need to construct mapping population; detect multiple loci at one time; high resolution.

The first research that utilized association mapping on plant genetics is in 2001 [4]. Several works have also been done on rice [5], maize [6], sorghum [7], wheat [8], barley [9], and potato [10]. As for cotton, a few studies have been carried out to understand its agronomic traits such as fiber traits and yield [11,12]. Some studies also are involved with branch traits [13–15].

In this study, the association mapping was carried out for 872 SSR markers of 39 lines and their 179 F_1_ hybrids in three environments. Different with previous studies on branch traits, the branches of cotton plant divided into three parts: upper, middle and bottom. This may help to find whether the branch traits of different parts have different genetic characteristics. PCR-based molecular markers SSRs (simple sequence repeats) from the cotton markers database (CMD) were extracted. Software *QTXNetwork* (http://ibi.zju.edu.cn/software/QTXNetwork/) was used in this study, which is based on mixed linear model approach [16]. And the association mapping was implemented by using a full genetic model with genetic effects of additive, dominance, epistasis and their environment interactions.

## Materials and Methods

### Parent selection and experimental design

A tested mapping population is representative for part of Upland cotton cultivars in China’s cotton-growing regions. These cultivars or lines **(**S1 Table) were provided by the Institute of Cotton Research, Chinese Academy of Agricultural Sciences and Tarimu University. The 39 cotton cultivars or lines (S1 Table) and their 178 F_1_ hybrids were analyzed by association mapping because of their genetic diversities. These materials were planted and evaluated for six branch traits in three environments (e_*1*_: Anyang in 2012; *e*_*2*_: Anyang in 2013; *e*_*3*_: Alar in 2012). A randomized complete block design with three replications was employed in the field trials. Each block was settled with two rows, and each row was kept in a 5.00 *m* long and 0.70 *m* wide plot. The distance between plants was 0.25 *m* wide (approximately 40 plants for each material in each replication) in Anyang; the distance between plants was 0.10 *m* wide (approximately 40 plants for each material in each replication) in Alar. Six fruit branch architecture traits were analyzed from random selected 10 plants for each material in each replication, these traits are bottom fruit branch node number (BFBNN), bottom fruit branch length (BFBL), middle fruit branch node number (MFBNN), middle branch length (MFBL), upper fruit branch node number (UFBNN), and upper fruit branch length (UFBL).

### DNA extraction and SSR markers screening

The leaves of cotton were collected in the pots in March 2012. Two cotyledons were taken from two plants of each variety (or line). DNA samples were extracted from 39 parents as described by Paterson *et al*. [17]. The downloaded sequences of SSR primes are mostly from the Cotton Marker Database (CMD, http://www.cottonmarker.org/cgi-bin/panel.cgi). This database contains all publicly available cotton SSR markers, which provides a more efficient utilization of molecular marker resources and will help accelerate basic and applied research in molecular breeding and genetic mapping in *Gossypium* spp. Polymorphic information is acquired from a standard screening panel including Upland cotton cultivars and other tetraploid species. A total of 5,052 SSR primer pairs were examined to screen for polymorphisms among thirty-nine inbred lines parents. These SSR primer pairs included CGR, GH, HAU, NAU, BNL, DPL, C and MGHES primer series. Marker nomenclature consisted of a letter indicating the origin of the marker, followed by the primer number. SSR analysis was conducted following the procedure described by Yao *et al*. [18]. For a difference stripe, if parent 1, 2 had no stripe, coded “-1” for the genotypes of SSR of parent 1, 2 (or *QQ* genotype); if parent 3, 4 had stripe, coded “1” for the genotypes of SSR of parent 3, 4 (or genotype *qq*), F_1_ from parent 1 and parent 2 is coded to “0”, F_1_ from parent 3 and parent 4 is coded to “1”, (or genotype *Qq*). For a couple of primer, only a stripe is coded for the same stripe to conducted association analysis. A total of 351 couple primers (872 difference stripes) showed polymorphic among the 39 Upland cotton varieties (or lines) in current study.

### Statistical analysis

The full genetic model for the phenotypic value of the *k*-th individual in the *h*-th environment (*y*_*hk*_) can be expressed by the following mixed linear model,

$$\begin{array}{c} y_{hk}=\mu +\sum_{i}a_{i}x_{A_{ik}}+\sum_{i}d_{i}x_{D_{ik}}+\sum_{i<j}aa_{ij}x_{AA_{ijk}}+\sum_{i<j}ad_{ij}x_{AD_{ijk}}+\sum_{i<j}da_{ij}x_{DA_{ijk}}+\sum_{i<j}dd_{ij}x_{DD_{ijk}}\\ +e_{h}+\sum_{i}ae_{ih}x_{AE_{ihk}}+\sum_{i}de_{ih}x_{DE_{ihk}}+\sum_{i<j}aae_{ijh}x_{AAE_{ijhk}}+\sum_{i<j}ade_{ijh}x_{ADE_{ijhk}}+\sum_{i<j}dae_{ijh}x_{DAE_{ijhk}}+\sum_{i<j}dde_{ijh}x_{DDE_{ijhk}}+\varepsilon_{hk} \end{array}$$

where *μ* is the population mean; *e*_*h*_ is the fixed effect of the *h*-th environment; *a*_*i*_ is the additive effect of the *i*-*th* locus with coefficient $u_{A_{ik}}$ (1 for *QQ*, coded *no stripe parent*; −1 for *qq stripe* coded *having stripe parent*); *d*_*i*_ is the dominance effect of the *i*-*th* locus with coefficient $u_{D_{ik}}$ (1 for *Qq*, 0 for *QQ* and *qq*, coded having stripe hybrid F_1_); *aa*_*ij*_, *ad*_*ij*_, *da*_*ij*_ and *dd*_*ij*_ are the digenic epistasis effects between the *i*-*th* locus and *j*-*th* locus with coefficients $u_{AA_{ijk}}$ (1 for *QQ × QQ* and *qq × qq*, −1 for *QQ × qq* and *qq × QQ*), $u_{AD_{ijk}}$ (1 for *QQ × Qq*, −1 for *qq × Qq*), $u_{DA_{ijk}}$ (1 for *Qq × QQ*, −1 for *Qq × qq*) and $u_{DD_{ijk}}$ (1 for *Qq × Qq*), respectively; *ae*_*ih*_ is the additive × environment interaction effect of the *i*-th locus in the *h*-th environment with coefficient $u_{AE_{ikh}}$; *de*_*ih*_ is the dominance × environment interaction effect of the *i-th* locus in the *h*-th environment with coefficient $u_{DE_{ikh}}$; *aae*_*ijh*_, *ade*_*ijh*_, *dae*_*ijh*_ and *dde*_*ijh*_ are the digenic epistasis × environment interaction effects between the *i*-*th* locus and *j*-*th* locus in the *h*-*th* environment with coefficient $u_{AAE_{ijkh}}$, $u_{ADE_{ijkh}}$, $u_{DAE_{ijkh}}$ and $u_{DDE_{ijkh}}$, respectively; and *ε*_*hk*_ is the residual effect of the *k*-*th* line or hybrid in the *h*-*th* environment.

### Association analysis

The SSR-trait association analyses were performed based on the above mixed linear model, and genetic effects were estimated by newly developed software QTXNetwork based on GPU parallel computation (http://ibi.zju.edu.cn/software/QTXNetwork/). The genotypic data (S1 Data CottonMeiYJ.Gen) and phenotypic data (S2 Data CottonMeiYJ.Phe) are included as a zip file (CottonMeiYJ.zip). All the detected SSR-trait association loci were fitted by a full model to estimate genetic effects of additive, dominance, epistasis as well as their environment interaction. The mixed linear model framework with Henderson method III [19] was used to construct the *F*-statistic test for the association analysis. 2,000 permutations of phenotypes were carried out to generate a null distribution of the extreme *P*-values. The most extreme *P*-value from each of the 2,000 scans was obtained and the 5% experiment-wise error rate was set for the 95% most extreme of these *P*-values. The QTS effects were predicted by using the MCMC (Markov Chain Monte Carlo) algorithm with 20,000 Gibbs sample iterations [20]. The distribution-based outliers were conducted for detecting and removing the outlier of phenotype. Based on the information of genetics effects for detected QTSs of six branch traits, the breeding values ($\hat{u}+\hat{e}+\hat{G}+\hat{GE}$) were predicted for the superior lines and superior hybrids of the mapping population [21].

## Results

### Estimated heritability and predicted genetic effects

Cotton has a within-canopy structure, and the fruit branches of plants can be divided into three parts: bottom, middle, and upper. The within-canopy structure of cotton is adjusted and affected by genetic basis and environment, and different part has different genetic inheritance rules. A total of 20 significant QTSs (*P*_EW_ -value < 0.05) were detected for six branch traits in cotton. The number of QTSs for each trait were 2 ~ 6, including DPL0061-2 for BFBL and MFBL, HAU2273-1 for MFBNN, BFBNN and BFBL. For the six fruit branch traits, estimated heritability for genetic effects was listed in Table 1. All the six branch traits were mainly controlled by genetic factors with high total heritability ($h_{T}^{2}\hat{=}\;52.85\;\sim \;93.45\%$). For BFBNN, dominance ($h_{D}^{2}\hat{=}\;43.98$) and epistasis ($h_{I}^{2}\hat{=}\;46.54$) were major genetic components. The dominance effects were also the primary genetic component for MFBNN ($h_{D}^{2}\hat{=}\;40.49$), MFBL ($h_{D}^{2}\hat{=}\;41.31$), UFBNN ($h_{D}^{2}\hat{=}\;47.36$), and UFBL ($h_{D}^{2}\hat{=}\;19.46$).

**Table 1:** Estimated heritability of QTSs detected for six branch traits.

Trait	$h_{A}^{2}$(%)	$h_{D}^{2}$(%)	$h_{I}^{2}$(%)	$h_{AE}^{2}$(%)	$h_{DE}^{2}$ (%)	$h_{IE}^{2}$(%)	$h_{T}^{2}$ (%)	*$R_{\hat{Y}}$*
BFBNN	1.09	43.98	46.54	0.79	1.82	1.78	96.00	93.45
BFBL	24.27	25.38	0.00	12.09	11.96	0.00	73.70	89.80
MFBNN	7.87	40.49	0.00	2.33	19.14	0.00	69.83	87.37
MFBL	21.02	41.31	11.18	1.95	7.98	0.00	83.44	72.48
UFBNN	14.93	47.36	0.00	4.38	0.71	0.00	67.38	63.83
UFBL	13.18	19.46	0.00	8.08	13.28	0.00	54.00	52.85

$h_{A}^{2}$ = heritability of additive effects, $h_{D}^{2}$ = heritability of dominance effects, $h_{I}^{2}$ = heritability of epistasis effects including *AA*, *AD*, *DA*, *DD*, $h_{AE}^{2}$ = heritability of environment-specific additive interaction effects, $h_{DE}^{2}$ = heritability of environment-specific dominance effects, $h_{IE}^{2}$ = heritability of environment-specific epistasis effects including *AAE*, *ADE*, *DAE*, *DDE*, $h_{T}^{2}$ = total heritability. $R_{\hat{Y}}$ = correlation coefficient between predicted values and phenotypic values.

Four QTSs were detected for BFBNN (Table 2) with highly significant QTSs (*P*_*EW*_-value < 1×10^−8^) (Table 2). CGR6795-1 had large positive dominance effect ($d\hat{=}\;1.165$, $h_{d}^{2}\hat{=}\;35.13\%$) but negative dominance × additive epistasis effect ($da\hat{=}\;-1.341$, $h_{da}^{2}\hat{=}\;46.54$). Increasing the BFBNN could be achieved by selecting genotype *Qq* of CGR6795-1 along with *Qq* of NAU879-1. HAU2273-1 also had positive effects of dominance ($d\hat{=}\;0.572$, $h_{d}^{2}\hat{=}\;8.48\%$) and additive effect ($a\hat{=}0.205$, $h_{a}^{2}\hat{=}\;1.09$).

**Table 2:** Predicted genetic effects, standard error, significance, and heritability for highly significant QTSs of six branch traits.

QTS	Effect	Estimate	SE	−Log*P*_*EW*_	*h*^2^ (%)
**BFBNN**					
CGR6795-1	*d*	1.165	0.082	45.0	35.13
HAU2119-1	*de*_*1*_	0.245	0.035	11.5	1.56
HAU2273-1	*a*	0.205	0.017	32.1	1.09
	*d*	0.572	0.061	20.4	8.48
	*ae*_*1*_	-0.096	0.020	6.1	0.24
CGR6795-1 × NAU879-1	*da*	-1.341	0.160	16.2	46.54
	*aae*_*1*_	-0.166	0.025	10.1	0.71
	*aae*_*3*_	0.373	0.060	9.3	3.61
**BFBL**					
CGR5534-3	*de*_*1*_	1.174	0.255	5.4	3.21
CGR6902-1	*a*	1.255	0.179	11.6	3.67
HAU1951-2	*a*	2.320	0.164	44.6	12.54
	*ae*_*1*_	-1.060	0.181	8.3	2.62
HAU2273-1	*a*	0.776	0.148	6.8	1.40
	*d*	3.155	0.525	8.7	23.19
	*de*_*1*_	2.498	0.545	5.3	14.54
HAU2469-1	*a*	1.514	0.194	14.2	5.34
	*ae*_*3*_	2.684	0.594	5.2	16.77
**MFBNN**					
HAU1385-2	*a*	0.229	0.020	29.7	7.47
	*ae*_*1*_	-0.157	0.022	11.6	3.54
HAU2273-1	*d*	0.477	0.069	11.2	32.57
**MFBL**					
BNL3348-1	*d*	-3.900	0.825	5.6	9.75
	*ae*_*1*_	1.292	0.251	6.6	1.07
	*de*_*1*_	4.233	0.919	5.4	11.48
CIR246-1	*a*	2.547	0.231	27.5	4.16
	*ae*_*1*_	-1.273	0.262	5.9	1.04
DPL0061-2	*a*	3.168	0.260	33.3	6.43
	*d*	-1.805	0.369	6.0	2.09
GH638-3	*a*	3.925	0.223	68.2	9.87
	*d*	6.245	0.705	18.1	24.99
HAU2119-1	*d*	2.644	0.396	10.6	4.48
DPL0061-2 × GH638-3	*aa*	-1.959	0.272	12.2	2.46
	*da*	1.955	0.389	6.3	2.45
**UFBNN**					
CGR5876-2	*a*	0.187	0.025	13.0	4.43
HAU2781-1	*a*	0.243	0.022	27.8	7.47
	*d*	-0.582	0.096	8.9	42.85
	*ae*_*1*_	-0.199	0.025	14.9	5.00
**UFBL**					
BNL4023-1	*a*	-1.222	0.255	5.8	2.17
	*d*	3.112	0.599	6.7	14.09
CGR6848-1	*a*	1.876	0.284	10.4	5.12
HAU1081-3	*a*	-1.934	0.269	12.2	5.44
HAU1434-1	*de*_*1*_	4.425	0.476	19.8	28.49

*a =* additive effect, *d* = dominance effect; *e*_*1*_ = Anyang in 2012, *e*_*2*_ = Anyang in 2013, *e*_*3*_ = Alar in 2012; *ae*_*1*_, *ae*_*2*_, *ae*_*3*_, *de*_*1*_, *de*_*2*_, *aae*_*1*_ and *aae*_*3*_ are the environment-specific genetic effect in given environment. −Log*P*_*EW*_ = minus log_10_(*P*EW−value).

A total of six QTSs were detected for BFBL (S1 Table), and five of these QTSs (except for DPL0061-2) had *P*_*EW*_-value lower than 1×10^−5^ (Table 2). Unlike other traits, additive effects were close to dominance effects, and environment-specific additive effects were also close to environment-specific dominance effects for BFBL. Positive additive effects were highly significant in four QTSs, and positive environment-specific dominance effects were also found for CGR6902-1 and HAU1951-2 in *e*_*1*_. In addition, HAU2273-1 was shared for increasing both BFBL and MFBNN by additive and dominance effects. In order to increase the BFBL, it was suggested to select genotype *QQ* of CGR5534-3, HAU1951-2 and HAU2469-1 with additive effect. Large positive environment-specific dominance of CGR6902-1 and HAU2273-1 can be selected for increasing BFBL of F_1_ hybrid in e_*1*_.

For two middle fruit branch traits, dominance effects were the main genetic effects, indicating that we can select high-performance offspring via hybrid especially in F_1_. MFBNN is found associated with two QTSs (S1 Table), and both of them were detected with additive, dominance and environment-specific additive effects in e_*1*_. HAU1385-2 had the environment-specific dominance effect and environment-specific additive effect both in *e*_*1*_ and *e*_3_.

HAU2781-1 was shared by BFBL and MFBNN both with additive, dominance and environment-specific additive effects in *e*_*1*_. During the breeding process, improving this trait could be obtained by selecting genotype F_1_ of HAU2273-1 with high-heritability dominance effect. Besides, selecting genotype *QQ* of HAU1385-2 could also increase the MFBNN especially in *e*_2_ and *e*_*3*_.

Six QTSs were significantly associated with MFBL (Table 2). Among them, four of these QTSs were involved in dominance effects. GH638-3 was detected with dominance effect, accounting for 24.99% of phenotypic variance. One pair of epistasis interaction DPL0061-2 × GH638-3 was identified with negative additive-additive and positive dominance-additive epistasis effects. Besides, two QTSs (HAU2119-1 and DPL0061-2) were shared by different traits. Selecting genotype *QQ* of CIR246-1 and DPL0061-2 could increase the MFBL by positive effects. Large positive dominance effects of two QTSs (GH638-3 and HAU2119-1) suggested that heterozygote *Qq* of these two QTSs could have heterosis of MFBL in F_1_ hybrids.

Topping is a vital step in cotton cultivating because there is apical dominance, which causes imbalance growth. From Tables 2 and 3 additive and dominance effects were main genetic components with not too large positive and negative effects. This showed that natural selection tended to control upper branch shorter than middle and bottom branches.

**Table 3:** Predicted genetic effects in 3 environments for genotype of *QQ*, *qq*, *Qq*, superior line, and superior hybrid for six branch traits.

Entry	*G*	*G+GE1*	*G+GE2*	*G+GE3*
**BFBNN**				
*QQ*	0.205	-0.057	-0.168	0.889
*qq*	-0.205	-0.274	-0.231	-0.143
Superior Line (+)	0.205	0.274	0.579	0.889
Superior Line (−)	-0.205	-0.274	-0.579	-0.889
F_1_	1.617	2.058	1.617	1.281
Superior Hybrid (+)	0.572	0.863	0.946	1.146
Superior Hybrid (−)	-0.501	-0.274	-0.579	-1.145
**BFBL**				
*QQ*	6.926	5.022	5.336	11.369
*qq*	-6.926	-5.022	-5.336	-11.369
Superior Line (+)	6.926	5.043	5.487	11.369
Superior Line (−)	-6.926	-5.043	-5.487	-11.369
F_1_	3.501	6.821	3.501	3.005
Superior Hybrid (+)	9.390	10.439	8.307	13.833
Superior Hybrid (−)	-7.008	-5.043	-5.569	-13.492
**MFBNN**				
*QQ*	0.281	0.051	0.281	0.419
*qq*	-0.281	-0.051	-0.281	-0.419
Superior Line (+)	0.281	0.091	0.281	0.419
Superior Line (−)	-0.281	-0.091	-0.281	-0.419
F_1_	0.712	1.165	0.712	0.269
Superior Hybrid (+)	0.712	1.165	0.712	0.843
Superior Hybrid (−)	-0.281	-0.091	-0.281	-0.419
**MFBL**				
*QQ*	8.614	8.278	8.614	8.441
*qq*	-12.532	-12.197	-12.532	-12.360
Superior Line (+)	8.614	8.278	8.614	11.594
Superior Line (−)	-12.532	-12.197	-12.532	-15.513
F_1_	3.184	8.701	3.184	-1.032
Superior Hybrid (+)	17.731	18.392	17.731	19.308
Superior Hybrid (−)	-16.433	-12.197	-16.433	-22.053
**UFBNN**				
*QQ*	0.211	-0.071	0.372	0.034
*qq*	-0.211	0.071	-0.372	-0.034
Superior Line (+)	0.649	0.534	0.588	0.827
Superior Line (−)	-0.649	-0.534	-0.588	-0.827
F_1_	-0.321	-0.192	-0.321	-0.321
Superior Hybrid (+)	0.696	0.710	0.696	0.827
Superior Hybrid (−)	-0.989	-1.072	-0.927	-1.166
**UFBL**				
*QQ*	-1.831	-1.831	-4.612	1.155
*qq*	1.831	1.831	4.612	-1.155
Superior Line (+)	5.583	5.583	4.612	8.569
Superior Line (−)	-5.583	-5.583	-4.612	-8.569
F_1_	5.823	10.248	3.032	5.823
Superior Hybrid (+)	8.190	12.615	6.501	11.176
Superior Hybrid (−)	-5.583	-5.583	-5.583	-8.569

*QQ* = homozygote of all loci with major-alleles; *qq* = homozygote of all loci with minor-alleles; *Qq* = heterozygote of all loci with *Qq*; Superior line = predicted genotypic effect of line in the selected population with highest values. Superior hybrid = predicted genotypic effect of hybrid in the selected population with highest values.

Four QTSs were found (Table 2) for UFBNN and two with highly significant QTSs (P_*EW*_-value < 10^−8^) (Table 2). CGR5876-2 and HAU2781-1 were both found with additive effects. HAU2781-1 also had dominance effect with considerable heritability of 42.85%. HAU1434-1 was shared by UFBNN and UFBL with additive, dominance and environment-specific dominance effects in *e*_*1*_, respectively. The UFBNN will grow when the breeders select genotype *QQ* of CGR5876-2 with positive additive effect. Furthermore, negative dominance of HAU2781-1 can be selected for decreasing UFBNN of F_1_ hybrid.

A total of four QTSs were detected for UFBL (S2 Table), and all of them were highly significant (Table 2). All these QTSs were found with additive effect. Except for CGR6848-1, other three QTSs had also dominance effects. And HAU1434-1 had environment-specific dominance effect in *e*_*1*_ and *e*_2_. No epistasis effects were detected in these two traits. Increasing UFBL of F_1_ hybrid could be selected by large positive environment-specific dominance of HAU1434-1 in *e*_*1*_ or BNL4023-1 for different environments. Selecting genotypes *QQ* of CGR6848-1 or *qq* of HAU1081-3 could increase UFBL.

### Predicted genetic effects for different genotypes

Besides the genetic effects of each QTS, the maximum positive and minimum negative genotypic effects of the superior lines and superior hybrids were also predicted in three environments (Table 3). For all the six fruit branch traits, predicted breeding values of the superior hybrids (+) were larger than the superior lines (+), which suggested that breeders could modify these traits through selecting F_1_ hybrids.

In the meantime, the genotypic effects of homozygotes (*QQ*, *qq*), and heterozygote (*Qq*) also were predicted for four traits in three environments. For MFBNN, MFBL and BFBL, the predicted genotypic effects were positive for major-allele homozygote *QQ*, but negative for minor-allele homozygote *qq*. It indicated that the natural selection tends to increase the middle branch node number and length, and also bottom branch length. This tower-like pattern is an ideal plant type for manipulating the plant density, enhancing the total light energy efficiency of a population, and increasing the yield [22]. As for UFBNN, no obvious pattern was discovered. Besides, the genotypic effects of *QQ* of BFBNN, BFBL, MFBNN and UFBL in *e*_*3*_ are significantly larger than them in *e*_*1*_ and *e*_*2*_. It may suggest that these traits would change in different environments and the environment in Alar is inclined to improve these traits. In Anyang, the high-yield variety is more tower-like. However, under the high-density condition in Alar, the middle and bottom fruit branch length need to be shorter and node number need to be less, and the upper fruit branch length needs to be longer and node number to be more. Thus, only by selecting different genotypes can breeders get high yield in different environments.

## Discussion

In this study, software *QTXNetwork* is applied to perform association mapping. This software has been successfully utilized in the previous study in cotton yield traits [23]. A full genetic model is used for estimating genetic effects of additive, dominance, epistasis and their environment interaction. Compared with a reduced model, a full model could retrieve the missing heritability and increase the total heritability. It could also help the researchers for better understanding the genetic basis and network in cotton branch traits. In this study, dominance, epistasis and their environment interaction were the major contribution of total heritability in each trait. The strong heterosis of cotton fruit branch traits was mostly due to these non-additive effects.

The calculated correlation coefficient between phenotype values and predicted genotypic effects of six traits ($R_{\hat{Y}}=\;0.53\;\sim \;0.93$) were close to the total heritability (Table 1). It was suggested that the full genetic model could predict the phenotypic variation very well. Furthermore, the heritability of BFBNN, MFBNN, and UFBNN was in descending order, which was also applicable to BFBL, MFBL and UFBL. This order indicated that the bottom branches were more stabilized during the inheritance process.

Several loci associated with the fruit branch traits were also detected by other researches on genetic mapping: CGR5876 [24]; GH220 [25]; HAU1385 [23]; GH638 [26]. These evidence verified that the mixed linear model approach was effective to reveal related loci of complex traits in cotton. Besides, seven QTSs involved in controlling more than one trait were detected. DPL0061-2 had positive additive and environment-specific dominance effects in *e*_*1*_ both for BFBL and MFBL, which suggested that common genes could improve the branch length by selecting the genotype *QQ* of DPL0061-2 in *e*_*1*_. The HAU2273-1 had positive additive and dominance effects for MFBNN, BFBNN and BFBL, indicating that improvement of branch node number and length could be obtained by selecting the genotype *QQ* or *Qq* of this QTS. Moreover, HAU1434-1 was shared by UFBNN and UFBL, including negative additive and positive dominance effects. This phenomenon indicated that some QTSs associated with upper fruit branch traits tend to maintain the tower-like plant shape.

## Conclusion

Association mapping is a statistically powerful method, which is utilized to dissect the genetic architecture of complex traits with high resolution. This study indicated that full genetic model, which included additive, dominance, epistasis and their environment interaction, could successfully predict the performance of causal loci. In total, 20 QTSs were significantly associated with six fruit branch architecture traits. And the results showed that dominance and epistasis effects played a significant role in the cotton fruit branch architecture traits. Marker assistant selection (MAS) breeding in different environments could obtain further improvements.

## Supporting Information

S1 Data Genotype data of CottonMeiYJ.Gen. (GEN)

S2 Data Phenotype data of CottonMeiYJ.Phe. (PHE)

S1 Table The material and origin of 39 Upland varieties (lines). (DOCX)

S2 Table Detected significant QTSs (*P*_*EW*_-value < 0.05) and predicted effects for six branch traits in cotton. (DOC)

## Abbreviations

BFBL: bottom fruit branch length
BFBNN: bottom fruit branch node number
CMD: cotton markers database
MAS: marker-assisted selection
MCMC: Markov chain Monte Carlo
MFBL: middle fruit branch length
MFBNN: middle fruit branch node number
QTLs: quantitative trait loci
QTSs: quantitative trait SSRs
SSR: simple sequence repeat
UFBL: upper fruit branch length
UFBNN: upper fruit branch node number
