# Supplementary material for: Dissecting Genetic Network of Fruit Branch Traits in Upland Cotton by Association Mapping Using SSR Markers
Source: PLoS One. 2017 Jan 25;12(1):e0162815. doi: 10.1371/journal.pone.0162815 (PMC5266336; doi:10.1371/journal.pone.0162815)
Supplement: S1 Table — (DOCX) [file pone.0162815.s003.docx]

**S1 Table. The material and origin of 39 Upland varieties (lines)**

| No. | Name | No. | Name | No. | Name | No, | Name |
| --- | --- | --- | --- | --- | --- | --- | --- |
| 1 | D10 | 11 | 279-28 | 21 | Xlz31# | 31 | Xlz67 |
| 2 | D14 | 12 | Kelin1918b | 22 | Cri49 | 32 | 223-23 |
| 3 | D17 | 13 | X-3 | 23 | 339-23 | 33 | 246-6 |
| 4 | Cri10 | 14 | Zaof | 24 | G3-2 | 34 | Xlz37 |
| 5 | Cri16 | 15 | 0-15 | 25 | Yumian15 | 35 | 325-1 |
| 6 | L7 | 16 | 0-19 | 26 | 29-2 | 36 | Jiyou768 |
| 7 | Tm-1 | 17 | 0-35 | 27 | 338-1 | 37 | 154 |
| 8 | L9 | 18 | Lu28 | 28 | Jimian20 | 38 | 155 |
| 9 | L11 | 19 | D9 | 29 | Xlz13 | 39 | 233 |
| 10 | L17 | 20 | Xlz23# | 30 | 337-17 |  |  |
