# Supplementary material for: Dissecting Genetic Network of Fruit Branch Traits in Upland Cotton by Association Mapping Using SSR Markers
Source: PLoS One. 2017 Jan 25;12(1):e0162815. doi: 10.1371/journal.pone.0162815 (PMC5266336; doi:10.1371/journal.pone.0162815)
Supplement: S2 Table — (DOC) [file pone.0162815.s004.doc]

**S2 Table. Detected significant QTSs (*P_EW_*-value < 0.05) and predicted effects for six branch traits in cotton**

| QTS | Effect | Predict | SE | -Log*P_EW_* | $h^{2}$(%) |
| --- | --- | --- | --- | --- | --- |
| **BFBNN** (Mean = 2.21) |  |  |  |  |  |
| CGR6795-1 | *d* | 1.165 | 0.082 | 45.0 | 35.13 |
|  | *de_1_* | 0.196 | 0.098 | 1.3 | 0.33 |
| HAU2119-1 | *ae_2_* | -0.174 | 0.053 | 3.0 | 0.61 |
|  | *ae_3_* | 0.201 | 0.061 | 3.0 |  |
|  | *d* | -0.120 | 0.031 | 4.1 | 0.37 |
|  | *de_1_* | 0.245 | 0.035 | 11.5 | 1.49 |
|  | *de_3_* | -0.336 | 0.084 | 4.2 | 1.49 |
| HAU2273-1 | *a* | 0.205 | 0.017 | 32.1 | 1.09 |
|  | *ae_1_* | -0.096 | 0.020 | 6.1 | 0.18 |
|  | *ae_3_* | 0.110 | 0.053 | 1.4 |  |
|  | *d* | 0.572 | 0.061 | 20.4 | 8.48 |
| CGR6795-1 × NAU879-1 | *aae_1_* | -0.166 | 0.025 | 10.1 | 1.78 |
|  | *aae_2_* | -0.200 | 0.055 | 3.6 |  |
|  | *aae_3_* | 0.373 | 0.060 | 9.3 |  |
|  | *da* | -1.341 | 0.160 | 16.2 | 46.54 |
| **BFBL** (Mean = 14.51) |  |  |  |  |  |
| CGR5534-3 | *a* | 0.574 | 0.180 | 2.9 | 0.77 |
|  | *ae_1_* | -0.585 | 0.196 | 2.5 | 0.27 |
|  | *d* | -0.656 | 0.234 | 2.3 | 1.00 |
|  | *de_1_* | 1.174 | 0.255 | 5.4 | 4.31 |
|  | *de_3_* | -2.042 | 0.860 | 1.8 |  |
| CGR6902-1 | *a* | 1.255 | 0.179 | 11.6 | 3.67 |
| DPL0061-2 | *a* | 0.486 | 0.174 | 2.3 | 0.55 |
|  | *d* | 0.571 | 0.248 | 1.7 | 0.76 |
|  | *de_1_* | 0.586 | 0.266 | 1.6 | 0.27 |
| HAU1951-2 | *a* | 2.320 | 0.164 | 44.6 | 12.54 |
|  | *ae_1_* | -1.060 | 0.181 | 8.3 | 3.28 |
|  | *ae_3_* | 1.759 | 0.588 | 2.6 |  |
| HAU2273-1 | *a* | 0.776 | 0.148 | 6.8 | 1.40 |
|  | *ae_1_* | 0.660 | 0.167 | 4.1 | 0.34 |
|  | *d* | 3.155 | 0.525 | 8.7 | 23.19 |
|  | *de_1_* | 2.498 | 0.545 | 5.3 | 4.85 |
| HAU2469-1 | *a* | 1.514 | 0.194 | 14.2 | 5.34 |
|  | *ae_1_* | -0.919 | 0.225 | 4.4 | 8.21 |
|  | *ae_2_* | -1.589 | 0.475 | 3.1 |  |
|  | *ae_3_* | 2.684 | 0.594 | 5.2 |  |
|  | *d* | 0.431 | 0.210 | 1.4 | 0.43 |
|  | *de_1_* | -0.938 | 0.226 | 4.5 | 2.54 |
|  | *de_3_* | 1.546 | 0.722 | 1.5 | 0.00 |
| **MFBNN** (Mean = 2.50) |  |  |  |  |  |
| HAU1385-2 | *a* | 0.229 | 0.020 | 29.7 | 7.47 |
|  | *ae_1_* | -0.157 | 0.022 | 11.6 | 2.08 |
|  | *ae_3_* | 0.137 | 0.066 | 1.4 | 0.00 |
|  | *d* | 0.235 | 0.077 | 2.7 | 7.92 |
|  | *de_1_* | 0.453 | 0.105 | 4.8 | 19.14 |
|  | *de_3_* | -0.443 | 0.105 | 4.6 |  |
| HAU2273-1 | *a* | 0.053 | 0.020 | 2.1 | 0.40 |
|  | *ae_1_* | -0.073 | 0.023 | 2.9 | 0.26 |
|  | *d* | 0.477 | 0.069 | 11.2 | 32.57 |
| **MFBL** (Mean = 20.96) |  |  |  |  |  |
| BNL3348-1 | *ae_1_* | 1.292 | 0.251 | 6.6 | 0.89 |
|  | *ae_3_* | -1.576 | 0.629 | 1.9 |  |
|  | *d* | -3.900 | 0.825 | 5.6 | 9.75 |
|  | *de_1_* | 4.233 | 0.919 | 5.4 | 7.62 |
|  | *de_3_* | -4.217 | 1.731 | 1.8 |  |
| CIR246-1 | *a* | 2.547 | 0.231 | 27.5 | 4.16 |
|  | *ae_1_* | -1.273 | 0.262 | 5.9 | 0.35 |
| DPL0061-2 | *a* | 3.168 | 0.260 | 33.3 | 6.43 |
|  | *ae_1_* | 0.641 | 0.292 | 1.6 | 0.09 |
|  | *d* | -1.805 | 0.369 | 6.0 | 2.09 |
|  | *de_1_* | 1.284 | 0.413 | 2.7 | 0.35 |
| GH638-3 | *a* | 3.925 | 0.223 | 68.2 | 9.87 |
|  | *ae_1_* | -0.996 | 0.255 | 4.0 | 0.63 |
|  | *ae_3_* | 1.404 | 0.630 | 1.6 |  |
|  | *d* | 6.245 | 0.705 | 18.1 | 24.99 |
| HAU2119-1 | *a* | 0.933 | 0.252 | 3.7 | 0.56 |
|  | *d* | 2.644 | 0.396 | 10.6 | 4.48 |
| DPL0061-2 × GH638-3 | *aa* | -1.959 | 0.272 | 12.2 | 2.46 |
|  | *ad* | 3.127 | 0.885 | 3.4 | 6.27 |
|  | *da* | 1.955 | 0.389 | 6.3 | 2.45 |
| **UFBNN** (Mean = 2.33) |  |  |  |  |  |
| CGR5876-2 | *a* | 0.187 | 0.025 | 13.0 | 4.43 |
| GH220-1 | *a* | -0.108 | 0.025 | 4.6 | 1.47 |
|  | *ae_1_* | -0.084 | 0.028 | 2.6 | 0.29 |
|  | *d* | 0.104 | 0.039 | 2.1 | 1.35 |
| HAU1434-1 | *a* | -0.111 | 0.026 | 4.9 | 1.57 |
|  | *ae_2_* | 0.161 | 0.068 | 1.7 | 2.42 |
|  | *ae_3_* | -0.177 | 0.068 | 2.0 |  |
|  | *d* | 0.158 | 0.039 | 4.3 | 3.15 |
|  | *de_1_* | 0.130 | 0.042 | 2.7 | 0.71 |
| HAU2781-1 | *a* | 0.243 | 0.022 | 27.8 | 7.47 |
|  | *ae_1_* | -0.199 | 0.025 | 14.9 | 1.67 |
|  | *d* | -0.582 | 0.096 | 8.9 | 42.85 |
| **UFBL** (Mean = 18.35) |  |  |  |  |  |
| BNL4023-1 | *a* | -1.222 | 0.255 | 5.8 | 2.17 |
|  | *d* | 3.112 | 0.599 | 6.7 | 14.09 |
| CGR6848-1 | *a* | 1.876 | 0.284 | 10.4 | 5.12 |
|  | *ae_2_* | -2.781 | 0.739 | 3.8 | 8.08 |
|  | *ae_3_* | 2.986 | 0.766 | 4.0 |  |
| HAU1081-3 | *a* | -1.934 | 0.269 | 12.2 | 5.44 |
|  | *d* | 1.443 | 0.480 | 2.6 | 3.03 |
| HAU1434-1 | *a* | -0.552 | 0.281 | 1.3 | 0.44 |
|  | *d* | 1.269 | 0.427 | 2.5 | 2.34 |
|  | *de_1_* | 4.425 | 0.476 | 19.8 | 13.28 |
|  | *de_2_* | -2.791 | 1.178 | 1.8 |  |

*a =* additive effect, *d* = dominance effect; *e_1_* = Anyang in 2012, *e_2_* = Anyang in 2013, *e_3_* = Alar in 2012; *ae_1_*, *ae_2_*, *ae_3_*, *de_1_*, *de_2_*, *aae_1_* and *aae_3_* are the environment-specific genetic effect in given environment. −Log*P_ER_* = minus log_10_(*P*_EW_ –value).
